# Implementing Integrated Care in Practice – Learning from MDTs Driving the Integrated Care Programme for Older Persons in Ireland

**DOI:** 10.5334/ijic.4682

**Published:** 2021-03-18

**Authors:** Sarah Barry, Maebh Ní Fhallúin, Stephen Thomas, PJ Harnett, Sara Burke

**Affiliations:** 1 Centre for Health Policy and Management, School of Medicine, Trinity College Dublin, Ireland; 2 Integrated Care Programme for Older Persons, Clinical Strategy and Programmes Division/Social Care Division, Health Service Executive, Kerry General Hospital, Tralee, Kerry, Ireland

**Keywords:** MDTs, integrated care, older people, Ireland, implementation, health system reform

## Abstract

The importance of multidisciplinary teams (MDTs) as critical implementation drivers emerged from this case study conducted with three pioneer sites implementing integrated care for older persons in Ireland as part of the Integrated Care Programme for Older Persons (ICPOP). We describe the practices of MDTs learning to deliver integrated care in service delivery settings, including the framework, resourcing, strategies, challenges and barriers they encounter.

The study was conducted by a team of researchers in collaboration with ICPOP at both national programme and pioneer site levels. Qualitative methods of participant observation, workshopping, and documentary analysis were used to build a rich description, and using organisational and systems lenses identification of critical factors as both themes and resources for learning.

The case study suggests the MDT is an essential driver of integrated care delivery. For example, ICPOP MDTs working across pioneer sites develop new service models and care opportunities, troubleshoot and challenge the systemic status quo, and disrupt professional silos. However, they also deliver on programme goals.

Nonetheless, progress is constrained by organisational factors including fragmented funding structures, high turnover of senior level decision-makers, a lack of multiannual funding and complex professional arrangements.

This study finds ICPOP offers practical and timely insight to inform health system reform. It embraces the complexity of delivery at national, local and community levels. The MDT emerges as an essential mechanism to manage such complexity and deliver on wider reform goals such as patient-centredness and timely access.

## Introduction

Integrated care is an important concept in the design and delivery of population, acute, primary and community care given the changes to health demographics in medium to high-income countries [1]. It means optimising early intervention and timely access to care in the community [2], and an emphasis on social supports and person-centeredness [3] – all complex service delivery goals. Ireland was late to commit to a comprehensive integrated care policy, only formally adopting one from 2018 as part of its ten-year Sláintecare Reform Programme mapping a path to universal healthcare [4,5,6].

This case study provides a valuable opportunity to examine early development of integrated care in Ireland. As it emerged as key to the case, the study also seeks to explore the MDT as a critical implementation driver of integrated care. In order to set this project in the broader context of whole-of-system change and healthcare reform in general, an organisational factors lens is used to analyse the case data and identify themes for discussion, reflection and learning.

**Figure 1:**
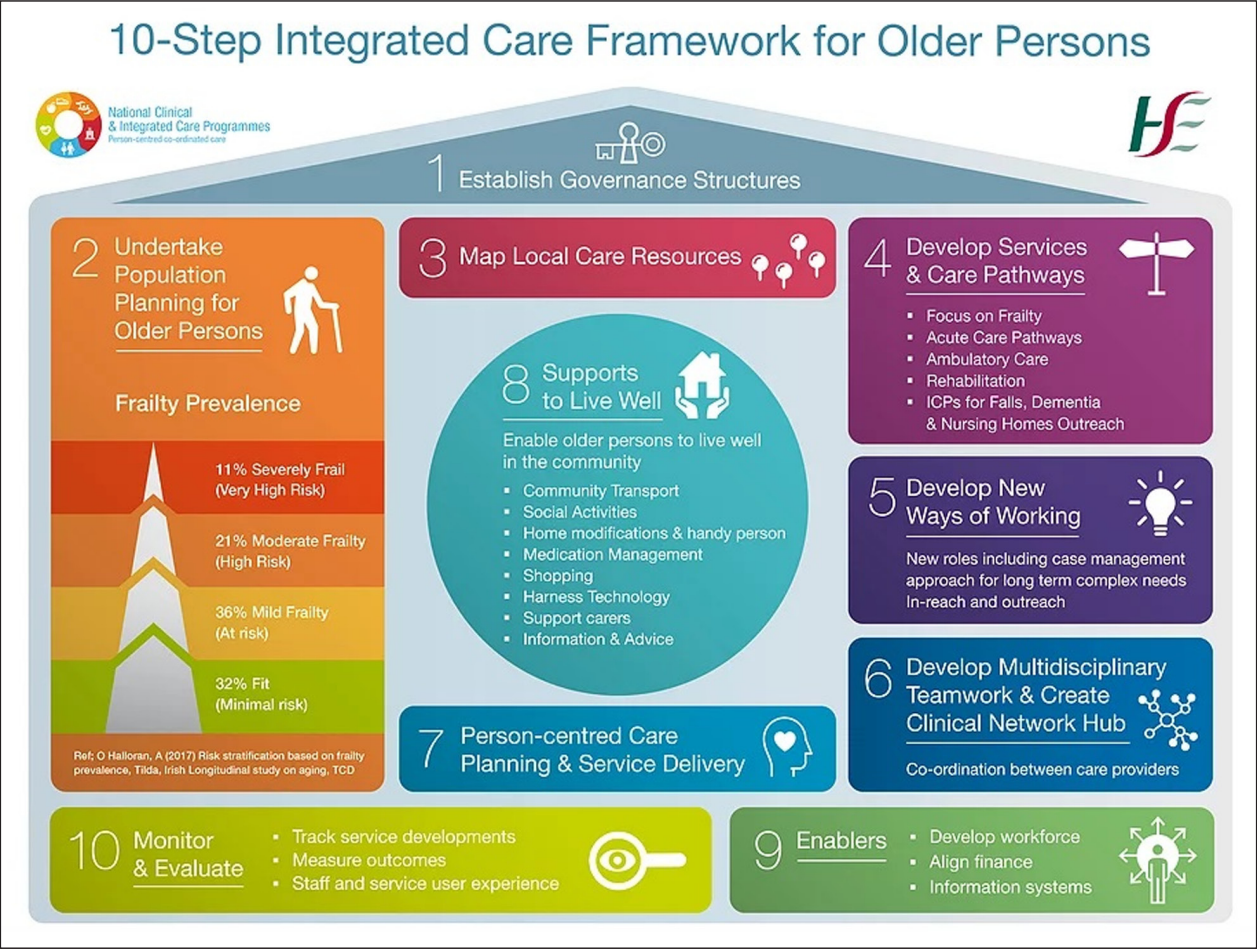
10-Step Integrated Care Framework for Older Persons.

ICPOP, as a national programme of the Health Service Executive (HSE) in Ireland was established in 2016 to improve the health of older people with complex care needs, with the primary objective of supporting them at home [7,8]. Funding was allocated on a phased basis to 13 pioneer sites located around the country between 2016 and 2018 to establish community-based MDTs and bespoke care pathways. After scoping and evidence-building [7,9] the sites began implementing a ‘10-step Integrated Care Framework for Older Persons’ in collaboration with patients, carers and local agencies. Implementation included developing new local governance structures, redefining professional roles and creating innovative ‘bottom-up’ work practices (see ***Figure 1*** for the 10-step Framework) [7 p. 292, 8].

This case study captures early-stage experiences of three pioneer sites after their first year of programme implementation with the aim of identifying useful learning for integrated care at delivery at service and system levels, and identifying critical themes to inform national health system-level reform.

## Methodology

The research team and ICPOP adopted a case study approach to highlight the descriptive and organisational elements of integrated care implementation in practice [10,11]. The lived experience of a representative selection of MDTs at work in their respective pioneer site locations was explored through participant observation, interviews, documentary analysis, and a workshop offering opportunity for reflection on initial findings, and respondent validation.

Participant observation as the primary data generation method was chosen to highlight practical and common ways of working [12]. The process of participant observation in the pioneer sites – including spending time at hospitals, primary care centres, community hospitals and patients’ homes – enabled shared experience of the working environment and offered insights into the real-life challenges of implementing change.

Observations were conducted by three researchers from the research team, with one researcher conducting the main body of on-site work. Interviews were conducted and transcribed by one researcher, with content analysis crosschecked by a second researcher.

Pioneer sites (A, B, C) were selected for the study based on:

1. Stage of ICPOP implementation, i.e. at least one year implementing the 10-step Framework
2. ICPOP MDT sponsorship type – case sites covered all three types, i.e.
  - Led by clinician in hospital and manager in community
  - Led by clinician in community and manager in community
  - Led by manager in hospital and manager in community
3. Geographic and ICPOP type mix- case sites were representative of all site types, i.e.
  - Rural/urban connected to model 4 hospital
  - Rural/urban connected to model 3 hospital
  - Urban connected to model 4 hospital

The selection was made by the ICPOP national team in collaboration with the pioneer sites and the research team after sites that met the above criteria were invited to participate in the research project. Of the sites meeting the criteria, those included volunteered participation to drive their own learning, and contribute to ICPOP evidence building. Feasibility and opportunity for participant observation was also a consideration in identifying the case study sites.

Participant observation was augmented with collection and analysis of operational and governance documents from each pioneer site. Based on these data a ‘site description’ was prepared and given to each participating MDT as a practical resource for team reflection and as a research tool. Discussion of site descriptions for respondent validation and transparency was generated through a workshop event in which findings were presented and discussed with MDTs and all attending ICPOP participants.

Site descriptions were developed through content analysis of case data materials by grouping case texts, closely reviewing materials and using MS Excel matrices and narrative text building to categorise findings. As themes emerged, they were discussed and agreed by the research team in order to identify a range of generalisable topics [10]. Given the focus on the day-to-day experience of MDTs, we aimed to identify organisational factors influencing how MDTs implement the ICPOP 10-Step Framework in practice.

This research was conducted with ethics approval from the Health Policy and Management and Centre for Global Health Research Ethics Committee (HPM/CGH REC) at Trinity College Dublin as part of the HRB-funded Pathways to Universal Health Care in Ireland Project (HRA-2014-HSR-499). Standard protocols were followed including using participant information leaflets, consent forms, data protection and assurance of anonymity.

## Results

In this section, we describe how engagement with case sites took place and how the process of data analysis proceeded; we also present in summary the ‘site descriptions’ produced. These describe the contexts and constituent factors of each site. Finally, we outline findings from the organisational factors analysis conducted. The themes explored highlight a range of specific implementation factors characterising the experiences of MDTs and ICPOP in general.

### Engagement with case sites and site descriptions

One research team member spent up to three days in each pioneer site, spending time with MDT members and observer participating in MDT activities, including meetings, patient consultations and domiciliary visits, and availing of opportunistic conversations with individual team members. Characteristics of locations, work settings, participants, and their interactions were documented by hand.

During and after the process of participant observation, documentary analysis of materials provided by pioneer sites relating to governance structures (including meeting agendas and minutes, terms of reference and presentations) was conducted. Secondary data sources, such as demographics of target populations, were also analysed to build a sense of the service context. Furthermore, seven semi-structured interviews were conducted with senior stakeholders including clinician and non-clinician managers.

All digitised notes were collated and chronologically ordered for each site. The site description was reviewed repeatedly as new information was added to generate an emerging understanding of the integrated care programme in the service context through the characteristics of each pioneer site. Initial content analysis of this data was conducted to identify the main challenges experienced by MDTs in each site, as well as their perceived strengths. Results were coded using MS Excel and distilled in discussions among research team members. They were then shared with the case sites as ‘site descriptions’.

These descriptions were presented as initial findings to all 13 pioneer sites at an *Integrated Care Network Day Workshop* attended by 89 ICPOP participants. The workshop presented an important opportunity to ensure respondent validity and transparency of findings. Participants from each of the three case sites discussed their own site descriptions with the research team. Between four and seven MDT team members from each site participated. Feedback was also received in writing from MDT members after the networking day and site descriptions amended accordingly. This iterative process provided opportunity to refine, distil and validate findings.

### Pioneer site descriptions

Detailed descriptions of the practice, resourcing and organisation of integrated care in the three case sites was developed (see ***Table 1*** for summary). Each site had a physical hub and a geriatrician led MDT. In all three sites, the governance structure established two project sponsors (in principle one from acute care and one from social care) to manage and lead coordination and progress. Established at different times over the three years before the study, all case sites received different levels of funding prior to ICPOP. The descriptions presented below reflect a snapshot of the different site service and operations contexts.

**Table 1:** Summary of three pioneer site descriptions.


	SITE A	SITE B	SITE C

**Catchment – Geography**	Urban & large rural area	Urban & large rural area	Suburban

**Catchment – Population demographic**	Older	Significantly older	Young but growing older population

**Hub Location**	Day hospital for older people on the grounds of a model 4 acute hospital	Community hospital close to a model 3 acute hospital	Primary care centre close to a model 4 acute teaching hospital

**Pioneer Site Background**	Green-field site (few services for older people outside of GP)	Evolved from integrated care activities for older people in the acute hospital	Creation of a community geriatrician post as catalyst

**Project Sponsors** **C = clinician** **nc = non-clinician**	Hospital Geriatrician (c) Older person’s services manager (nc)	Acute Hospital General Manager (nc) Community Health Organisation (CHO) Head of Social Care (nc)	Consultant Geriatrician (Former community geriatrician) (c) CHO Head of Social Care (nc)

**The Programme funded MDT Members**	Senior Physiotherapist Senior OT Administrator Clinical Nurse Specialist Social worker	Clinical Nurse Specialist for Dementia Senior Grade Occupational Therapist Senior Grade Physiotherapist Administrator	Consultant Geriatrician (rotates every 4 months) Clinical Case Manager × 2 (one to be filled) Senior Occupational Therapist Senior Physiotherapist Senior Social Worker Administrator

**Referrals**	From GP and acute hospital	From acute and community hospital	From GP and acute hospital

**Domiciliary Visits**	Provided by social worker	Home visits and assessment provided by physio and OT	Domiciliary visits undertaken by all members of the team

**Governance**	Weekly MDT meetings. Steering Committee meets quarterly; Working groups (for ambulatory care, rehabilitations and early mobilisation) meet quarterly	Weekly MDT meetings. Steering Committee meets bi-monthly; Implementation Team meets bi-monthly.	Weekly MDT meetings. Steering Group meets every two months. Multidisciplinary business meetings held monthly.

**Outreach Activities**	GP educational meetings; roadshow to raise awareness among public health nurses, presentation at national Integrated Care Conference	Stakeholder planning workshop including patient advocates to map existing services and to set priorities for the year	Presentations to GPs, Nurses, at Integrated Care Conference, Attendance at Age Friendly County Alliance, Relationships built with Alzheimer’s Day Centres/services

**Next Steps**	Secure funding for a dietitian, psychologist, pharmacist, speech and language therapist, and a community geriatrician.	Increase ICPOP services, scope supports for nursing homes (esp. for dementia patients), develop end of life care, frailty and delirium education and training	Support long term care residents through the development of a nursing home liaison service and recruit a dietitian


#### Site A

The hub in Pioneer Site A is located in a day hospital for older people serving an urban and large rural area and located on the grounds of a Model 4 acute hospital. (A Model 4 hospital in Ireland provides 24/7 acute surgery, acute medicine, critical care, tertiary care and, in certain locations, supra-regional care).

Referrals to the MDT/ICPOP coordinated services come from GPs and hospitals. Prior to ICPOP, services for older people developed incrementally and sequentially without an overarching design. In this case, the crisis closure of hospital rehabilitation beds due to under-resourcing served as a shock or ‘focusing event’ to galvanise and unite acute and community workers to improve services for older people.

The MDT collates data on discrete project impacts that are returned to the ICPOP national team. They have also teamed up with the local university to pilot a common digital repository for patient assessments. Team members put significant energy into communications and outreach. For example, they undertook a roadshow to raise awareness of ICPOP services among public health nurses. They have no community geriatrician; therefore, the social worker is the only team member who provides domiciliary visits. The MDT experienced some early-stage operational issues around triage and defining what should be considered a “crisis case”.

#### Site B

The hub in Pioneer Site B is in a community hospital located close to a model 3 acute hospital (Model 3 hospitals provide 24/7 acute surgery, acute medicine, and critical care). Their catchment area has an urban and rural population significantly older than the national average. The MDT has funding for a community geriatrician and a case manager but neither positions were filled at the time of the research. A wide geographical service catchment area contributes to the recruitment problem with long travel times for home visits. Two new advanced nurse practitioners had recently joined the MDT, which accepts hospital referrals only resulting in little GP or public health nurse involvement. Home visits and assessments are provided by a physiotherapist and an occupational therapist. Strong links exist between the community and acute hospitals, and a few team members have extensive institutional knowledge.

#### Site C

The hub in Pioneer Site C is based in a suburban primary care centre close to a large model 4 acute hospital. This is the only team of the three sites with a specifically named ‘community geriatrician’ and dedicated case manager in post. The community geriatrician is a 4-month rotating position that gives all registrars the opportunity to work with the ICPOP MDT.

There are interesting glimpses of evolving interdisciplinary approaches in site C, where health and social care professionals have identified common tasks around case management. Site C has developed its own common assessment tool.

Referrals are accepted from GPs and the hospital, and domiciliary visits are undertaken by all members of the team. Efforts are being made to raise awareness of services among GPs, nurses and community groups. Funding has been secured for a nursing home liaison case manager. A lot of time is spent on communication with patients and health, social and community care partners, the value of which may not be captured by current performance indicators.

### Analysis of Organisational Factors Influencing Implementation

An organisational lens was used to identify and examine the factors influencing MDT implementation of integrated care in practice under the ICPOP 10-Step Framework. These include MDT membership, skills, competencies, new opportunities, and leadership. Likewise, organisational processes such as reporting structures, evolving roles and common language and tools are explored. From a systems perspective factors tempering implementation in terms of the broader environment are also highlighted, these include programme design limitations, the 10-Step Framework and contextual, situation-based challenges.

Some factors were evident in one site while others form part of a broader pattern visible across all three sites. The issues highlighted below are considered important by all respondents for successful implementation of integrated care and scaling ICPOP at a system level as confirmed during the workshop event in which findings were reported and discussed.

#### Skills & competencies

The main competency of successful ICPOP MDTs is trust. Weekly meetings where referred patient cases are discussed indicate teams where members are well acquainted with one another, particularly in the rural settings. Relationships are long established resulting in open and highly participatory team meetings. One MDT member described them as “the most powerful tool”.

The geriatrician or case manager generally leads discussion and all members are invited to contribute to build a patient and carer, or family profile. Members demonstrate a deep respect and understanding of the human condition of ageing. In some cases, where skills and experience allow, they enable patient autonomy in managing their own care and carrying responsibility for the possible risks involved. Indicating the importance of the skill-mix designed for ICPOP hub and site teams, the sites where most core MDT positions are filled display a high level of energy and enthusiasm among members and in meetings.

#### Team membership and new opportunities

The excitement of being a pioneer site, of having the freedom to innovate according to local interpretation of the 10-step Framework, having a sense of purpose, a shared goal and the support of a tight-knit team is perceptible across all three case-sites. Communication with other MDT members and with multiple professional and non-professional partners is a significant energiser of members’ new roles despite a possible lack of role-specific training. Team members value opportunities for exchanging ideas and approaches at events such as the ICPOP Network Days and through regular communication with the programme level service-improvement managers.

#### Leadership and established professional boundaries

Case site sponsors highlight the importance of team members having an “integrated care ethos” as well as an understanding of the current system and a willingness to learn from other team members. Many MDT members demonstrate leadership qualities, such as courage, the skills and competencies required to innovate, communicate, build networks and nurture relationships with a wide variety of partners. Sites rely heavily on individual personalities to drive strategy and innovation – which is a leadership strength, but potentially also a weakness that needs to be managed systemically.

In response to this potential challenge, in one site (Site A) members of the MDT spend a lot of time consulting with and communicating with colleagues and the public about their work. They use conversation, visual communications and presence in acute and community settings to build broader understanding and buy-in to the integrated care approach.

The outcome seems to be a good understanding of the causes of resistance to change and greater overall buy-in among colleagues. On the importance of earning trust among professional colleagues, one member stated, “they know they are not going to be shafted” by the MDT; this comment refers to common fears around caseload management and duplication of roles as integrated care is implemented. As a reassurance to colleagues regarding professional boundaries, members in one site strongly expressed they are “not trying to replace primary care”.

#### Reporting structures

MDT reporting structures vary across case sites with some members reporting to line-managers within their discipline, and others to a MDT lead outside their discipline. In one site, an allied health professional reports to their professional line manager who is not directly involved with ICPOP.

To mitigate confusion this manager is invited to monthly business meetings to be informed of ICPOP activities. While presenting potential challenges in practice this locally arranged structure has the benefit of expanding the ICPOP approach to a broader professional network. In another site, the health and social care professionals on the MDT report to a community manager who is not a member of the team. Some members self-select to be part of integrated care teams, while others are transferred from other areas of the HSE.

This variation in reporting structures reflects the localisation capacity of the ICPOP 10-Step Framework as a strength, whilst also highlighting a potential weakness for the sustainability of MDTs that do not enjoy a unified vision on line management arrangements.

#### Evolving roles and the burden of changing the care model

Role definition and management is an on-going challenge for MDTs and their members. Roles are based on newly designed job descriptions, including those of the community geriatrician, advanced nurse practitioner and case manager. While members are required to develop their roles according to ICPOP goals and the needs of their patients, many are not involved in strategic planning for the pioneer site and some are unclear about the source of funding for their roles, whether from the community or the acute budget.

Team members expressed concerns relating to how their performance is being measured without factoring for example how their new roles are different from what they were trained to do. For example, they find they spend more time now on communications and administration rather than direct contact with patients. Related to this issue of less contact-time with patients, some team members expressed concern about how their colleagues in traditional professional roles view them (including their line managers) given the innovative nature of integrated care delivery.

Fears of losing specialist skills due to working in the community were also apparent. Despite freedom to innovate with the 10-step Framework, there is a sense among team members that many of the factors relevant to integrated care implementation through the MDT are outside their control. As demand for services exceeds the capacity of the MDT to deliver care, managing the number of patients accessing ICPOP services is an important issue.

One member outlined how MDTs have “the power to remove fiefdoms” however; professional identification and hierarchy remain strong in all three sites. Another team member reported that working in the community is viewed as “dumbing down” or “going native” by some hospital-based clinicians.

Some members expressed feeling like outliers in their professions, detached from their colleagues because of their ICPOP involvement. One consultant doctor highlighted the need for task shifting, “we need nurse-led and therapist-led clinics; we will get more value for the medical intervention then”. Existing organisational and professional structures and relationships, between hospital, community, GP and primary care, have a strong impact on the scope and level of ambition of all teams trying to develop new ways of working. Also of note is the fact that MDTs in all sites comprise of more females than males, possibly due to established gender profiles across the professions incorporated into MDTs, but nonetheless highlighting a challenge in ensuring diversity in integrated care implementation.

#### Common language and tools

MDTs have a strong desire to achieve integrated care for older persons however; there are different understandings about what successful integrated care looks like. Conversations about “integration” indicate evidence of systems thinking at clinical levels, however, it is not clear if such integrated understanding extends to broader organisational, professional, or national levels.

One member managed this challenge by boxing the problems of the health system as too great. They feel the best response is to be effective by focusing on local issues by developing an interdisciplinary approach. This local focus evolving in one site for example as is evident as health and social care members identify and share common tasks around case management.

Additionally, new pathways for frailty, falls and dementia care delivery, and task shifting or sharing provide evidence of other innovative interdisciplinary approaches. Discussion is also taking place in the case sites on the potential introduction of a Standardised Assessment Tool (SAT). These and other initiatives suggest how by developing common understanding, language and practical tools, MDTs can drive or “transform” the care delivery model.

#### Programme design limitations

MDT members are aware of the limitations of the targeted nature of ICPOP in treating older patients with complex care needs only (5% of older population). Recognising the need to provide services for “the 95%”, one member stated, “we will never be able to offer everyone frailty treatment, primary prevention of frailty is key”.

Despite this some MDT members feel there will always be a need for a specialist service like that which ICPOP currently provides. While the service is currently consultant-led, one senior clinician pointed out that a specialist could be a non-medic defined as an advanced-practitioner as is already the practice in parts of the UK.

There is a sense of disempowerment among pioneer site members regarding issues they perceive to be outside their control and that impact adversely on their work. These include a lack of home care services, lack of data and electronic records, the use of out-of-date protocols, underdeveloped primary care, and existence of pre-defined indicators to measure progress (mostly quantitative). On this point, one team member commented, “discharged could mean dead”.

#### The 10-Step Framework

Senior managers report positively on the usefulness of the 10-Step Framework, which was designed by ICPOP as an evidence-based guide for pioneer sites to develop new integrated care structures, work practices and care pathways [9]. The challenge however of developing new pathways for patients with different needs is highlighted by one team member noting how “potential patient pathways are hugely complex, even people working in services find it difficult to navigate them”. Reflecting the difficulty of matching services to individual patient needs, one member commented, “Some patients don’t fit the system”.

Given the emergence of the importance of MDTs as key drivers of integrated care, the research team decided to read the case data in the broader context of implementing programmatic or system-level change on the assumption that MDTs need systemic support. With this in mind, several factors categorised into local, programme and system levels were identified. This stage of analysis leads to a discussion of findings and conclusions in the final section of this paper.

Numerous organisational and systemic challenges associated with MDT operation, which to a greater or lesser degree influences the pace of ICPOP implementation in each site, are identified. The following key factors were identified as having a particularly strong influence in all three sites (See ***Table 2***). Exploring these factors, at local, programme, and system level indicates how naturally supportive of change a setting is and the likelihood of successfully achieving integrated care as envisaged in the 10-Step Framework.

**Table 2:** Factors influencing programme implementation progress.


Local Level Factors

Socio-Demographics Geography (rural/urban difference) Legacy health system issues Existing physical structures and organisations Existing relationships and links between individuals and institutions Personalities Community resources Culture and beliefs (including language) Local political and economic factors

Programme Level Factors

Workforce and leadership capacity Power structures and hierarchy

System Level Factors

National political and economic factors Fragmented funding structures Quick turn-over of senior level decision makers Lack of commitment to multiannual funding Lack of clarity on regional administrative boundaries Complex professional contractual negotiations Slow implementation of the national eHealth strategy.


For example, recruitment is difficult in rural settings where long travel times are involved. Areas with large populations of older people, or areas of socio-economic deprivation experience greater demand for older persons’ services. Some sites are green-field sites while others are “retrofitting” existing services and structures. Existing relationships between professionals and institutions are strong determinants of successful implementation. The lack of strategic involvement of GPs in ICPOP reflects fragmentation in the Irish health system, where GPs who are largely publicly funded operate independent private practices separate to the public system. Public health nurses as important community-based clinicians are not significantly involved either.

Other challenges relate to the policy context. A historical weakness of national policy to provide clarity on a future direction for health and social care services, integrated funding, and robust governance structures were identified in pioneer sites as a barrier to progress.

The 10-Step Framework, as a tool for guiding decisions is viewed as helpful, however it is clear implementation is constrained by organisational and system factors. These include, as noted in ***Table 2***, fragmented funding structures (e.g. between community and hospital care), quick turnover of senior level decision-makers, a lack of authority and commitment to multiannual funding, lack of clarity on regional administrative boundaries and complex professional contractual negotiations. Uncertainty around future funding for ICPOP, an inherent feature of such a pioneer programme, is a challenge for medium and long-term strategic planning and resourcing.

Slow progress implementing the national eHealth Strategy is also reported to be a constraint. Sites are currently heavily dependent on individuals with institutional knowledge, memory and experience in the absence of adequate data systems. One site has taken the step of teaming up with a local university to trial its own form of electronic health record and digital repository. It is not clear yet if this is likely to be scaled or whether it can be easily integrated with other local, regional and national health data systems.

## Discussion

Several generalisable themes emerge from the case study for discussion and learning. MDT innovation should be supported, building on team experience and professional expertise, as well as the opportunities created. For MDTs to successfully implement new practices they need systemic supports since their energy and enthusiasm for innovation, when it takes place *despite* the system, may reduce over time. MDT members offer a useful perspective for the system more broadly as a cross-disciplinary group of professionals with experience of changing practices. Nonetheless, clinicians trained in the biomedical model may benefit from training in systems thinking [13].

The challenges of moving through change, including uncertainty about roles, funding and programme sustainability, and the impact on professional standing due to cultural perceptions need to be formally addressed. Being a pioneer is difficult and understanding the fears of MDT members is important. Those fears relate to the inadequacy of performance measures, the disconnect between training and competencies for new roles, maintaining a manageable workload, the loss of specialist skills, colleagues’ perceptions, and uncertainty about the future of ICPOP. Pioneers often have to justify their funding in a competitive environment for resources, against other interventions that may have a clearer, evidence-based demand.

We also note the importance of programme branding and identification with a shared integrated care vision, as well as getting the right balance between centralised leadership and resourcing, and local adaptation of the 10-Step Framework. ICPOP developed a strong brand and a positive feeling among frontline staff. Communications and outreach activities, including framing messages to resonate with different groups, takes time and resources but appears to be effective in earning the trust of patients and colleagues.

Scaling up may require a strong brand to increase awareness, with adequate scope for local adaptation, interpretation and ownership of the model. New locally adapted work practices are more important than any single model. Programmes should seek synergistic activities with other programmes from the outset to ensure efficient use of resources. Continuous knowledge exchange will contribute to the development of a culture of creativity and experimentation and reduce unhealthy competition and exclusivity.

The issues of scaling a programme like ICPOP means taking a properly resourced population health approach. Currently ICPOP targets older people with complex care needs only. It is not clear if the longer-term goal is to create permanent MDTs dedicated to delivering integrated care for older people around the country or if their role is to embed integrated care into all services through the new Community Health Networks (CHNs) [14].

A population or mainstreaming approach would involve assimilating integrated care for older people into general services and ensuring all services are age-attuned and integrated. It will be important for health service senior management and planners to discuss long-term goals in order to manage staff and public expectations. If ICPOP is to be scaled from its current targeted programme focused on complex cases to include the 90–95% who require more basic frailty prevention, there will need to be sustained investment in clinical leadership and resourcing of MDTs in the community to deliver to this population.

Doing this in the context of a fragmented system is challenging, particularly given the public-private mix in Ireland.

In response, ICPOP builds partnerships across professions and institutions, harnessing goodwill and challenging frontline staff to develop creative solutions to problems. It is bringing hospital and community services and professionals closer together through joint leadership in the pioneer sites and joint funding of MDTs working across those settings. While still in early development, there are signs this programme could help shift Ireland’s approach to older peoples’ care from heavily medicalised and hospital-centric to a more holistic, community-based, social care approach.

ICPOP is trialling new community-based multidisciplinary interventions and adopting a case management approach to address the needs of older people with complex care needs. The 10-Step Framework appears to be an effective evidence-informed guide designed to support local interdisciplinary teams, providing a map of high-level milestones for pioneer sites without imposing a strict method of travel [7]. This allows pioneer sites to develop various models of integrated care according to local resources and need.

ICPOP’s national programme office has put significant energy into supporting pioneer sites with service improvement managers working across multiple sites providing advice and guidance. It has developed a communications and engagement strategy to raise awareness and promote co-produced service design. Its outreach activities include networking events for those involved to exchange ideas, approaches and lessons. The office is also collecting quantitative data on programme activities [15].

On this basis, ICPOP potentially provides a blueprint for combined top-down/bottom up approach to whole system change in that it supports the implementation of Sláintecare [6] and offers useful implementation learning more broadly. The challenges encountered by frontline staff and the system pressures highlighted through this case study need to be addressed for change to happen at scale.

For population-based integrated care, engagement with primary care providers is critical. ICPOP is led locally by hospital geriatricians and HSE managers working in the public health system. Yet GPs and public health nurses are the first point of contact in the community for most of the target population. While public health nurses are HSE staff, GPs are self-employed contractors.

The low level of GP and public health nurse involvement in ICPOP operation and governance is contradictory to the idea of integrated community-based care. This problem reflects a system-wide lack of integration between Ireland’s public and private service providers, including GPs, many health and social care professionals, hospitals and nursing homes. The challenge of integrating publicly and privately funded services to provide an integrated model of care remains largely unsolved.

ICPOP can be considered a micro-meso level intervention due to its focus on creating new care pathways and bridging the gaps between professions and organisations [16]. Research shows that while the goal of a community-based programme may not be to change the whole system, its activities can have system-wide effects and, therefore, it should be viewed in the context of the whole system [17,18]. This suggests that a micro-meso level programme could be considered a potential driver of system change.

In order for a pilot or pioneer site-based programme to achieve scale it requires clear national-level policy that sets the agenda for more proactive and collaborative care [19]. Meso- or micro-level activity should take place in a supportive policy environment. This does not necessarily mean imposing narrow interpretations of integrated care on pilots, which could restrict innovation; rather national policy-makers should use national policy to remove the barriers restricting progress.

Many of the barriers reported by MDTs in this study concern macro level issues, such as the lack of home care services and under-developed primary care. The lack of integration at the national system level is also negatively affecting implementation. Sláintecare, now providing a clearer policy context can facilitate progress through mutually reinforcing policies, goals and principles and supportive legislation where required [6].

## Conclusions

Some limitations inhibited the scope of this study. Given resource challenges a practical decision was taken to conduct participant observation in three pioneer sites (chosen for their representative nature) rather than engaging all 13 ICPOP pioneer sites that would have resulted in greater participation and a fuller picture. A further limitation is given the early stage of ICPOP implementation at the time of the study, and the related data gaps, consideration of outcomes including performance indicators and patient experience reports was not possible. The resulting focus on process outcomes [20] focusses the study on MDT experience. We hold, nonetheless given Ireland’s poor track-record of successful large-scale health and social care reform implementation, attention to process and organisational challenges is apt. [6,21,22].

ICPOP embodies a grounded approach to health system reform, embracing the challenges of complexity, translation to the local context and community engagement through design and delivery of integrated care for older persons by MDTs working at the frontline [9,15,23]. This focus on the lived experience and innovation of MDTs within the first year of ICPOP implementation adds insight and texture. Despite the early stage of implementation and the risks identified for ICPOP operation, we found evidence of effective MDT contribution at local and system levels. Nonetheless, for integrated care delivery be sustainable and scalable, MDTs cannot work in isolation. Organisational and system factors including fragmented systems, siloed funding and established professional boundaries should be addressed to facilitate innovative and effective delivery of integrated care.

Carrying out cases studies over a longer period and a formal evaluation, including patient outcomes and experience would provide further valuable insight and a stronger evidence base contributing to the implementation of integrated care, locally, regionally, nationally and internationally.
